# Facile preparation and characterization of a novel visible-light-responsive Rb_2_HgI_4_ nanostructure photocatalyst

**DOI:** 10.1039/d1ra03152j

**Published:** 2021-09-16

**Authors:** Elham Abkar, Mojgan Ghanbari, Omid Amiri, Masoud Salavati-Niasari

**Affiliations:** Institute of Nano Science and Nano Technology, University of Kashan P. O. Box. 87317-51167 Kashan Iran Salavati@kashanu.ac.ir +98 31 55913201 +98 31 55912383; Faculty of Chemistry, Razi University Kermanshah 6714414971 Iran; Department of Chemistry, College of Science, University of Raparin Rania Kurdistan Region Iraq

## Abstract

Visible photocatalytic procedures exhibit encouraging potential in water purification by increasing the photocatalytic performance. Therefore, the improvement of low-cost and efficient photocatalysts for environmental remediation is an increasing demand, and photocatalysts based on semiconductors have gained considerable attention due to their superior stability and activity. In the current study, novel Rb_2_HgI_4_ nanostructures were prepared *via* a simple, low-cost, and low-temperature solid-state method. The effects of different parameters such as type of surfactants, reaction temperature, and reaction time were studied on the structure, crystallinity, particle size, and shape of nanostructures. This new compound has a suitable band gap (2.6 eV) in the visible region. The photocatalytic performance of Rb_2_HgI_4_ was examined for the removal of coloring agents under visible light irradiation and it was found that this compound could degrade and eliminate acid black 1 by about 72.1%.

## Abstract 2

In this study, we reported Rb_2_HgI_4_ nanostructures as a new and efficient photocatalyst for the first time. We used the solid-state method for the fabrication of this compound at a low temperature.

## Graphical abstract

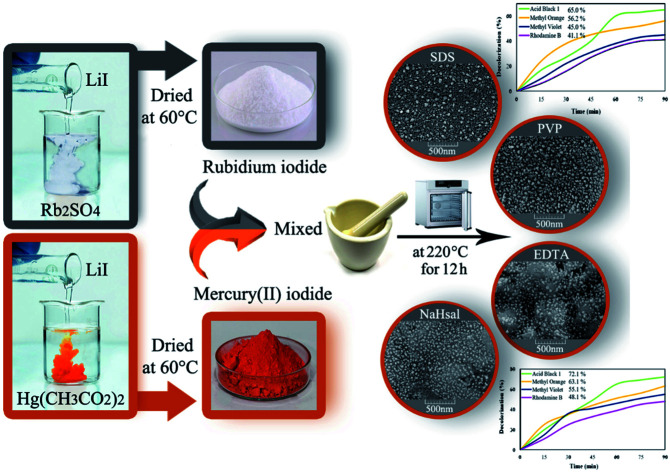

## 1. Introduction

Nowadays, the primary requirement for freshwater reservoirs has drawn worldwide attention due to fast development in industrialization, urbanization, and massive population growth. These accelerated developments have led to ecological issues, including polluted groundwater and air along with hazardous wastes. It is predicted that the growing demand for freshwater will worsen due to the continuous release of contaminants and pollutants in natural water sources. The reusing and recycling of sewage are essential to enhance the inadequate supply of freshwater.^1,2^ Numerous strategies, including biological treatment,^3^ adsorption,^4^ chemical treatment,^5^ and membrane-based separations^6^ have been studied to establish specific water purification. Biological approaches traditionally developed to efficiently eliminate the multiple varieties of pollutants from water finally led to the generation of secondary contaminants, including health-threatening bacteria and soluble refractory organic compounds, which are challenging to eliminate.^7^ Hence, the improvement of sustainable, nondestructive, and green technology for wastewater/water purification is of highest concern. Semiconductor-based photocatalysts are one of the most successful strategies for wastewater/water treatment owing to their high potential and high efficiency in eliminating toxic organic pollutants utilizing solar light.^8–11^ The structural, electronic, and optical properties of the material must be carefully studied to promote efficient semiconductor-based photocatalysts for the photodegradation of contaminants in wastewater/water. Commonly, various characteristics, for instance the selection of semiconductor materials, morphological architecture, and surface features, should be considered when designing efficient and stable visible-light-sensitive photocatalysts.^12^ To design more effective photocatalysts based on semiconductors, strategies based on fundamental principles have been a beneficial instrument in presenting a broad comprehension of photocatalysis, describing experimental data, and predicting innovative semiconductor photocatalyst materials with excellent performance.

Semiconductors, including CdS, ZnS, Fe_2_O_3_, ZnO, and TiO_2_, can be used as sensitizers for photoinduced redox reactions because of the electronic configuration of the metal atoms in the chemical composition, which is described with an empty conduction band (CB) and a filled valence band (VB).^13,14^ By radiation, VB electrons (e−) are directed to the CB producing holes (h^+^). These e^−^–h^+^ pairs can either interact separately with other molecules or can recombine. The holes might react with hydroxide ions or with electron donors in the solution to create strong oxidizing agents such as superoxide (O_2_^−^˙) or hydroxyl (˙OH) radicals.^15–17^ Alternatively, semiconductors are substances whose VB and CB are separated by band gap or energy gap. When a semiconductor receives photons with energy equivalent to or higher than its band gap, electrons in the VB can get excited and jump up into the CB, therefore producing charge carriers. The recombination of e^−^–h^+^ needs to be restricted as much as possible after the first charge separation to have an efficient photocatalytic reaction.^18^

Several studies have been conducted on halide ferroelectrics having the general formula A_2_BX_4_ (A = Rb, Cs, k, Tl; B = Hg, Cd, Zn, Co; X = Cl, Br, I).^19–22^ They are categorized into two groups with various structures. One group is ferroelectrics possessing an orthorhombic β-K_2_SO_4_ structure with the *Pmcn* space group, and another has a monoclinic Sr_2_GeS_4_ structure with the *P*2_1_/*m* space group.^23^ Rubidium tetraiodomercurate(ii) (Rb_2_HgI_4_) is one of these compounds that is categorized into two classes: superionic materials identified as solid electrolytes, and thermochromic materials, which are a branch of smart materials.^24^ Solid electrolytes provide the movement of ions without the need of a liquid. The cations in the compounds induce electrical conductivity with their motions.^25^ Superionic materials are intermediates of solid crystals with organized structure and liquid crystals without organized structure that possesses mobile ions. These compounds are employed as solid batteries, fuel cells, multiple chemical sensors, and supercapacitors. Today, ecological contamination has become a worldwide disaster; therefore, new energy reservoirs, including fuel cells and solar cells have drawn considerable attention. Photodegradation is also one of the most important technologies used in the disposal of pollutants in industrial wastewater. Researchers want to use natural resources available to obtain the energy needed for the degradation of dyes in industrial wastewater, and the most important source of natural energy is sunlight that consists of about 5–7% of UV light, 46% of visible light and 47% of infrared radiation.^26,27^ The photocatalytic oxidation of numerous harmful organic dyes in industrial wastewater has been carried out over different semiconductor photocatalysts under UV light irradiation. Research is now focused on achieving high photocatalytic efficiency with new photocatalysts, particularly with sunlight. Due to the suitable band gap of Rb_2_HgI_4_ (2.6 eV), we decided to study its photocatalytic activity under visible light for the first time. In this research, we have applied the solid-state method to produce Rb_2_HgI_4_ nanostructures. Our main purposes are noted below:

(1) Fabrication of Rb_2_HgI_4_ nanostructures by a facile route.

(2) Investigation of the photocatalytic activity of this compound for the first time.

Rb_2_HgI_4_ nanostructures were constructed *via* a simple, low-cost solid-state method at low temperatures to achieve the first aim. The morphology of the nanostructures was homogeneous, and we selected this pathway to produce the nanostructures and studied the influence of different types of surfactants on the morphology of the obtained products. So far, nano-Rb_2_HgI_4_ has not been reported, and this is the first time that Rb_2_HgI_4_ nanostructures are synthesized.

The as-synthesized Rb_2_HgI_4_ nanostructures were applied as photocatalysts for the first time to fulfill the second aim. In this study, we have used Rb_2_HgI_4_ nanostructures as a novel and efficient catalyst for the photodegradation of organic dyes.

## 2. Experimental

### 2.1. Materials

Each chemical reagent applied in the current study was supplied in excellent quality. Lithium iodide (LiI·2H_2_O), mercury(ii) acetate (Hg(O_2_CCH_3_)_2_), rubidium sulfate (Rb_2_SO_4_), ethylenediaminetetraacetic acid (EDTA), sodium dodecyl sulfate (SDS), sodium salicylate (NaHSal), and polyvinyl pyrrolidone (PVP-25000) were obtained from Merck Company and used without further purification. Acid black 1 (C_22_H_14_N_6_Na_2_O_9_S_2_), methyl orange (C_14_H_14_N_3_NaO_3_S), methyl violet (C_24_H_28_N_3_Cl), and rhodamine B (C_28_H_31_ClN_2_O_3_) were purchased from Sigma-Aldrich.

### 2.2. Preparation of precursors

The RbI precursor was fabricated by a facile co-precipitation method from Rb_2_SO_4_ and LiI. First, a certain amount of Rb_2_SO_4_, LiI, and different surfactants (such as EDTA, SDS, NaHSal, and PVP) was liquefied in distilled water in separate beakers, and the solutions were combined with each other. The resultant solution was allowed to stand for crystallizing the white RbI precipitate. An HgI_2_ precursor was synthesized by adding a LiI solution to Hg(O_2_CCH_3_)_2_ solution to obtain an orange precipitate.

### 2.3. Fabrication of Rb_2_HgI_4_

**Scheme 1:**
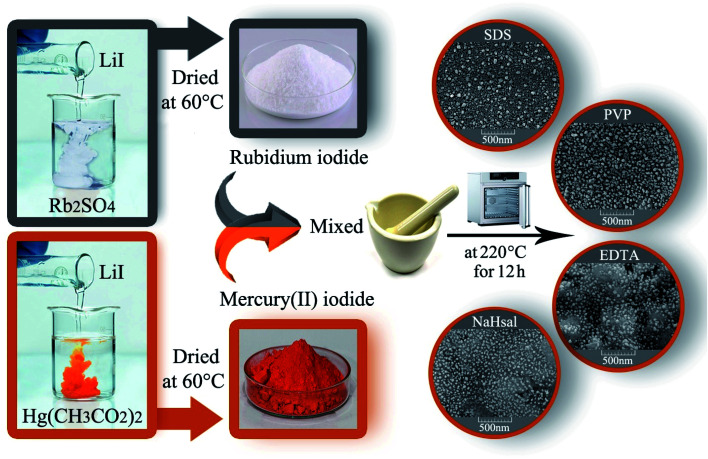
Schematic of the fabrication of Rb_2_HgI_4_ nanostructures.

HgI_2_ and RbI were mixed in a certain ratio and ground in a mortar. The resulting powder was heated at different temperatures (180–220 °C) in air for 9–12 h. Before the temperature reaches 220 °C, the average temperature rise is 5 °C per minute. After continuing the thermal treatment at 220 °C for a specified time, it was allowed to cool naturally to room temperature (Scheme 1). Table 1 indicates the different conditions for the fabrication of Rb_2_HgI_4_ to obtain the best conditions.

**Table 1:** Preparation conditions for Rb_2_HgI_4_

Sample no.	RbI : HgI_2_ molar ratio	Type of surfactants	Time (h)	Temperature (°C)	Grain size (nm)
1	2	—	12	220	38.8
2	2	SDS	12	220	33.5
3	2	PVP	12	220	39.4
4	2	EDTA	12	220	34.4
5	2	NaHsal	12	220	36.7
6	2	SDS	9	220	35.8
7	2	SDS	15	220	32.6
8	2	SDS	12	200	37.0
9	2	SDS	12	180	37.1

### 2.4. Photocatalytic performance

The photocatalytic activity of Rb_2_HgI_4_ was examined *via* its potential for the degradation of different organic colorants under visible radiation. An Osram light (150 W) was employed as the radiation source, containing a wavelength range of 400–780 nm for the photocatalytic process. The experiments were conducted without catalyst and light, and almost no dye was degraded after 90 min. 100 mg Rb_2_HgI_4_ was added to 100 mL 10 ppm of dye solution in each experiment. The suspension was mixed in dark for 0.5 h before turning on the visible light. 5 mL sample was removed from the suspension every 15 min during irradiation and centrifuged at 10 000 rpm for 4 min. The buoyant was collected, separated, and observed with a UV-Vis spectrophotometer.

## 3. Result and discussion

### 3.1. Characterization

**Fig. 1:**
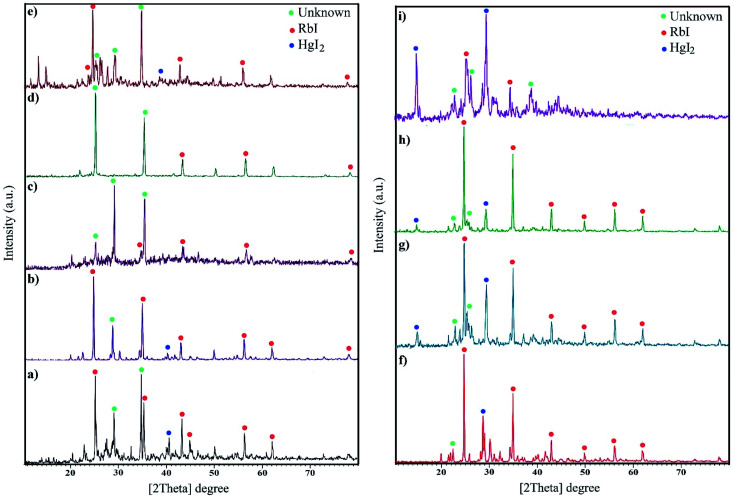
XRD pattern of samples (a) 1, (b) 2 (c) 3, (d) 4, (e) 5, (f) 6, (g) 7, (h) 8, and (i) 9.

The XRD patterns are fingerprints that can help us understand what is in compounds. The XRD pattern of a compound is a simple sum of the diffraction peaks of every phase. Fig. 1a indicates the XRD pattern of sample 1 with a molar ratio of 2 : 1 for RbI to HgI_2_ formed from rubidium iodide (JCPDS no. 01-073-0385) and mercury iodide (JCPDS no. 01-072-1615) and an amount of unknown phase. Fig. 1b–e presents the XRD patterns of samples in the presence of different surfactants at 220 °C for 12 h. A large amount of unknown phase is observed in these patterns in addition to HgI_2_ (tetragonal structure) and RbI (cubic structure). The remarkable feature in all XRD patterns is the presence of an unknown phase at different 2*θ* values that does not match with any of the combinations reported in the database. We aimed to fabricate Rb_2_HgI_4_ nanostructures; however, since the reference code of Rb_2_HgI_4_ has not been reported, we believe that the general pattern of Rb_2_HgI_4_ is related to these XRD patterns. Fig. 1f–i show the effects of reaction time and temperature on the purity of products. Reducing the time of the reaction to 9 h decreased the unknown phase (Fig. 1f). Alternatively, this phase did not have enough time to form. In contrast, increasing the time to 15 h caused an increase in the unknown phase (Fig. 1g). Decreasing the temperature to 200 °C and 180 °C reduces crystallinity and the unknown phase. According to the XRD patterns, the optimum condition was selected at 220 °C for 12 h in the presence of SDS as a capping agent. The crystallite size was determined by the Scherrer equation: *D* = *Kλ*/*β* cos *θ* (ref. 28) was found to be between 32 and 39 nm.

**Fig. 2:**
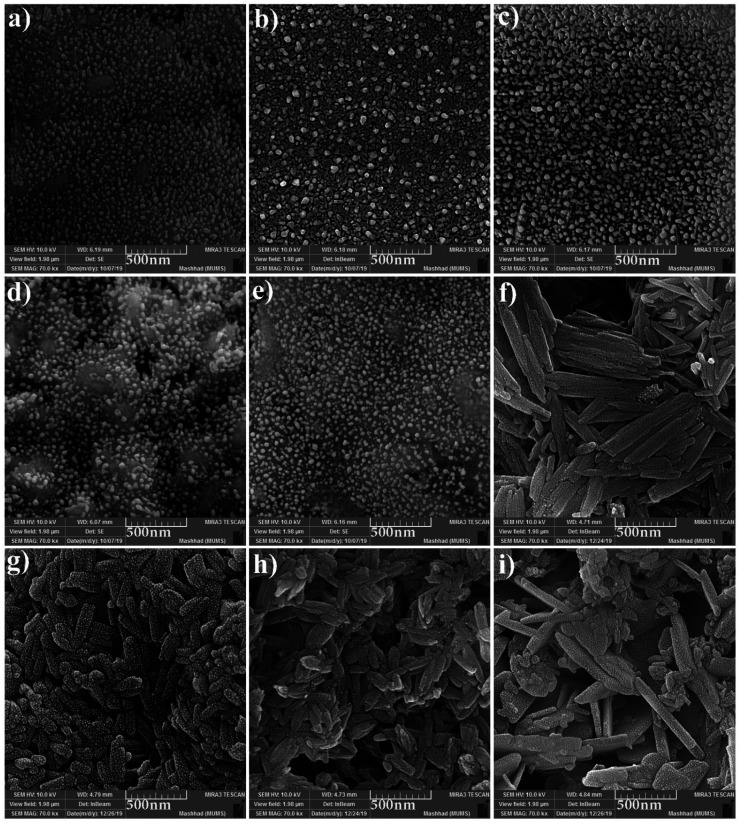
FESEM images of the samples (a) 1, (b) 2 (c) 3, (d) 4, (e) 5, (f) 6, (g) 7, (h) 8, and (i) 9.

**Fig. 3:**
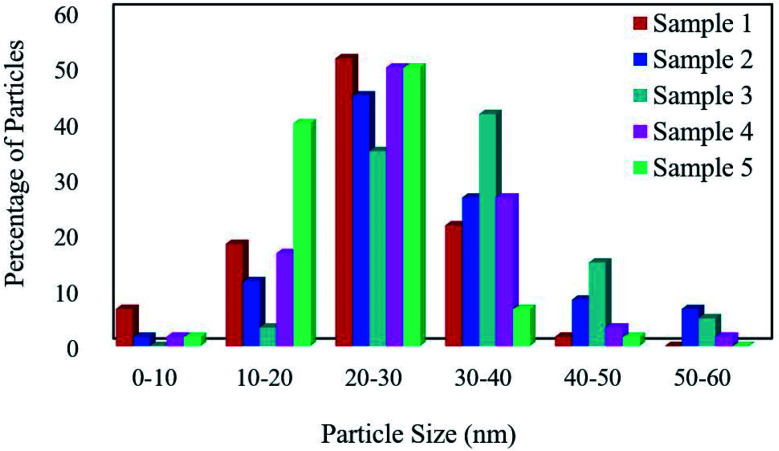
The particle size distribution of the samples.

The FESEM images of samples were obtained for studying the morphology, uniformity, shape, and particle size (Fig. 2). The average particle size was measured by the Digimizer software. Fig. 2a indicates that without surfactant, nanoparticles with an average particle size of 24 nm are formed at 220 °C for 12 h (sample 1). Fig. 2b–e show the effects of different types of surfactants. It can be seen that homogenous nanoparticles are formed by using all the surfactant types. Reducing the time of reaction to 9 h caused irregular rod-like structures and microstructures (Fig. 2f). Nanoparticles with an average size of 9 nm were formed on the microstructures by increasing the time of reaction to 15 h (Fig. 2g). Fig. 2h and i show that decreasing the reaction temperature to 200 °C and 180 °C resulted in bulk structures. Therefore, the optimum condition was selected in the presence of SDS as an anionic capping agent at 220 °C for 12 h. Fig. 3 displays the histogram size distribution of samples 1–5 obtained using the Digimizer software, indicating that most particles are between 20 and 30 nm.

**Fig. 4:**
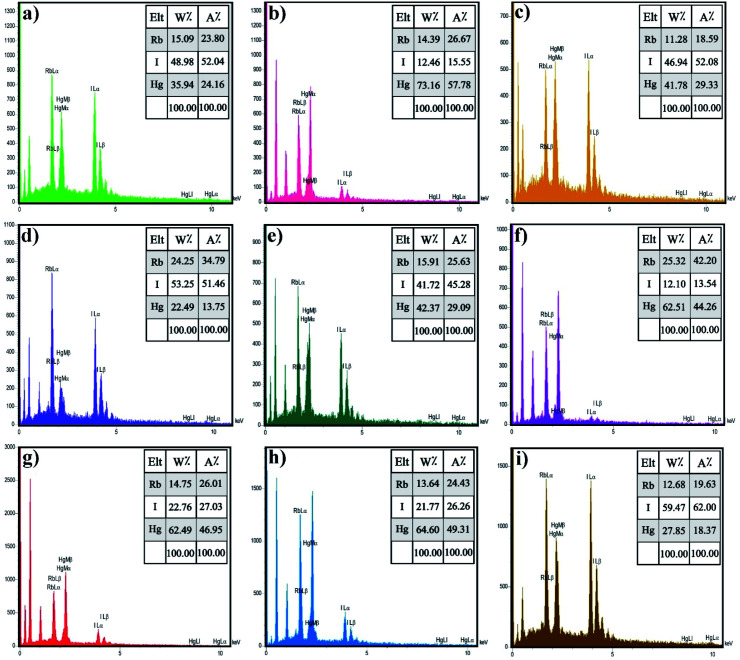
EDS spectrum of the samples (a) 1, (b) 2 (c) 3, (d) 4, (e) 5, (f) 6, (g) 7, (h) 8, and (i) 9.

Energy-dispersive X-ray spectroscopy (EDX) is an analytical method utilized for chemical characterization or elemental analysis of a sample. Fig. 4 demonstrates the EDX spectra of Rb_2_HgI_4_, which indicate that all the peaks are assigned to Rb, Hg, and I elements. Consequently, the products are perfectly purified and related to the XRD outcomes. Besides, the EDX result confirmed the uniform dispersion of the elements on the samples.

**Fig. 5:**
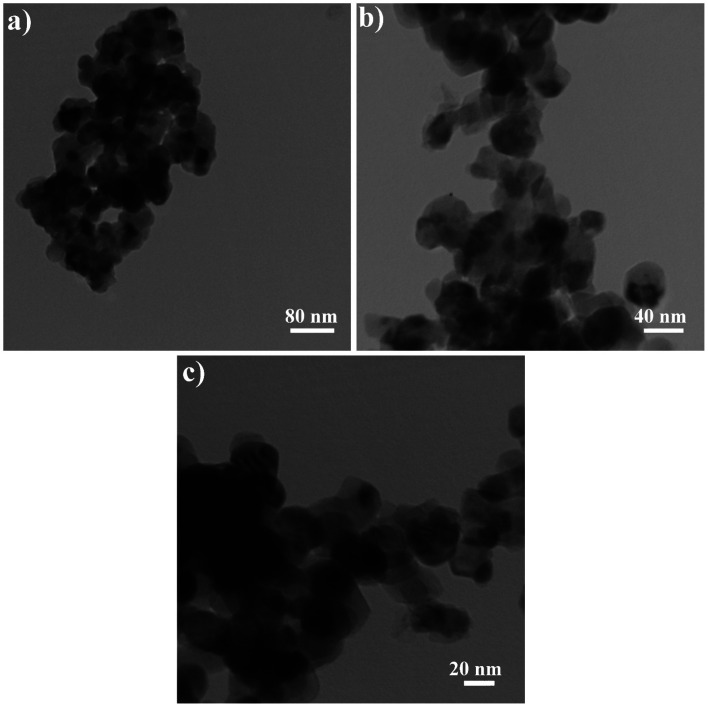
TEM images of sample 2.

Fig. 5 shows the TEM photographs of Rb_2_HgI_4_ nanostructures (sample 2) in different scales of 80, 40, and 20 nm. Uniform nanoparticles with an average size of 28 nm are observed in this figure, which corresponds to the SEM and XRD outcomes.

**Fig. 6:**
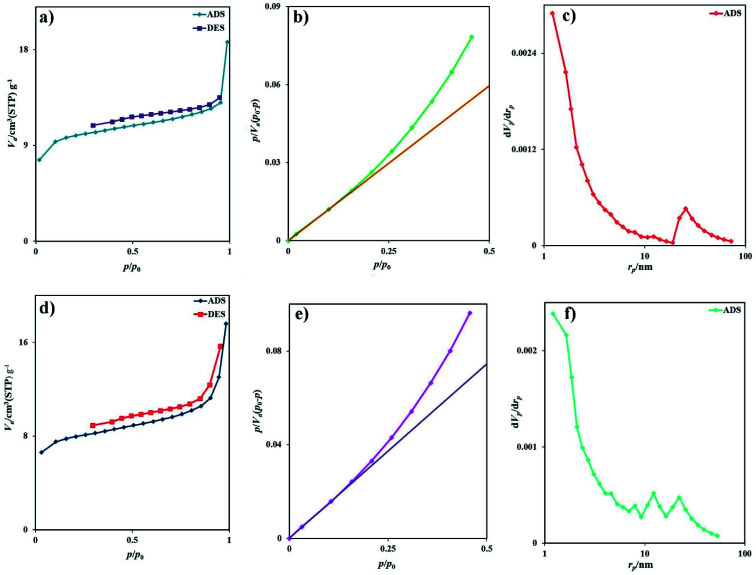
(a) Low temperature N_2_ adsorption/desorption isotherm, (b) BET plot, (c) BJH plot of sample 2, (d) low temperature N_2_ adsorption/desorption isotherm, (e) BET plot, and (f) BJH plot of sample 7.

The BET surface area analysis is a standard instrument to calculate the specific surface area and pore volume of samples. The morphology of samples prepared with different surfactant types is similar, and there is no need to analyze the BET surface area of all samples. The adsorption–desorption isotherms of nitrogen for samples 2 and 7 are depicted in Fig. 6a and d, respectively. Based on the IUPAC category, sample 2 exhibits the isotherm type III with the H4-type hysteresis loop (Fig. 6a), which is ascribed to the mesoporous materials (Fig. 6b). The specific BET surface area was calculated to be 36.6 m^2^ g^−1^, and the average pore diameter was 3.16 nm. From the BJH plot, the total pore volume and pore diameters were 0.0289 cm^3^ g^−1^ and 1.21 nm, respectively (Fig. 6c). The isotherm of sample 7 is type III with an H3-type hysteresis loop, indicating microporous materials (Fig. 6d). The specific surface area was calculated to be 29.2 m^2^ g^−1^, and the average pore diameter was 3.72 nm. From the BJH plot, the average pore volume and pore diameters are 0.027 cm^3^ g^−1^ and 1.21 nm, respectively (Fig. 6f). The specific surface area of sample 2 (*S*_BET_ = 36.6 m^2^ g^−1^) is greater than that of sample 7 (*S*_BET_ = 29.2 m^2^ g^−1^). This information is well corroborated with the SEM and TEM results. Table 2 compared the BET data of this nanostructure with other iodide nanostructures. Rb_2_HgI_4_ nanostructures have a large specific surface area compared with CuPbI_3_, Tl_4_HgI_6_, and Tl_4_CdI_6_ and possess a small average pore diameter.

**Table 2:** The surface characteristics related to iodide compounds

Sample	BET surface area (m^2^ g^−1^)	Pore volume (cm^3^ g^−1^)	Mean pore diameter (nm)	Ref.
Rb_2_HgI_4_ (sample 2)	36.6	0.029	3.16	This work
Rb_2_HgI_4_ (sample 7)	29.2	0.027	3.72	This work
CuPbI_3_ nanostructures	15.57	0.055	14.24	39
CuPbI_3_ nanostructures	10.49	0.010	3.87	39
TlPbI_3_/Tl_4_PbI_6_ nanocomposites	51.54	0.031	2.43	40
TlPbI_3_/Tl_4_PbI_6_ nanocomposites	24.20	0.020	3.28	40
Tl_4_HgI_6_ nanostructures	1.79	0.41	5.90	38
Tl_4_HgI_6_ nanostructures	1.16	0.27	12.39	38
Tl_4_Cdl_6_ nanostructures	5.84	0.108	9.68	29
Tl_4_Cdl_6_ nanostructures	5.39	0.124	36.34	29
CsPbl_3_ nanostructures	61.79	0.13	8.33	33
CsPbl_3_ nanostructures	68.74	0.10	6.05	33

**Fig. 7:**
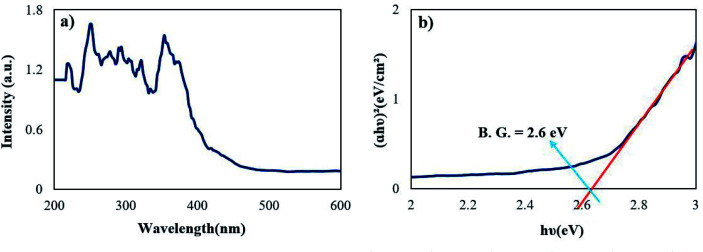
(a) DRS spectrum and (b) optical density (*αhν*)^2^*vs.* energy (*E*) plot of the samples 2.

Fig. 7 represents the optical properties of sample 2 *via* UV-Vis diffuse reflectance spectroscopy (DRS). The Rb_2_HgI_4_ nanostructure displays typical absorptions in the range of 200–400 nm. The band gap (B.G.) can be defined employing the following equation:^29^

1 *A*(*hν* − B.G.) = (*αhν*)1/*r*,

where, *A* is the material constant, hυ is the light energy, *r* is 2 or 1/2 for indirect and direct allowed transitions, and *α* is the absorption factor.^29^ Considering that a band gap is observed in the (*αhν*)^2^*vs. hν* diagram and this value does not match with the band gap of RbI^30^ and HgI_2_,^31^ it indicates the formation of a pure (not composite) compound called Rb_2_HgI_4_. The optical band gap of Rb_2_HgI_4_ was determined at 2.6 eV.

### 3.2. Photocatalytic activity

**Fig. 8:**
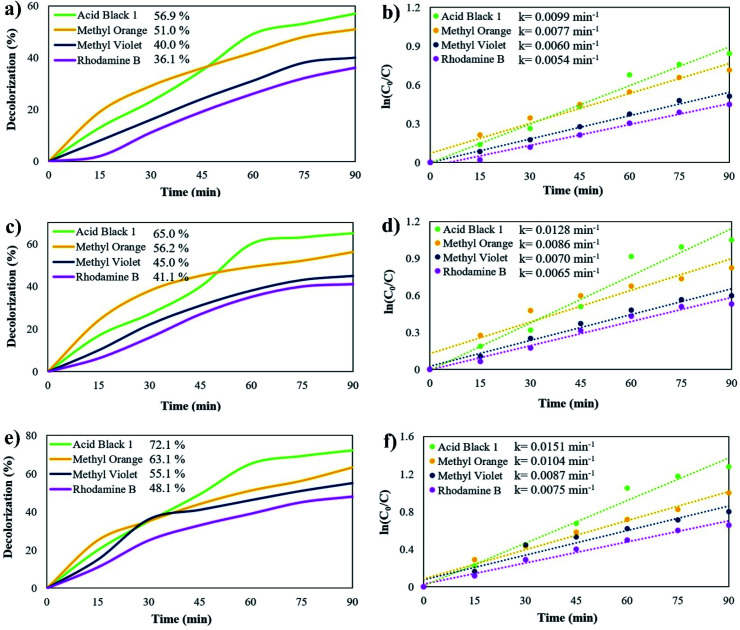
Photocatalytic degradation of different coloring agents over sample 2 with different dosages of Rb_2_HgI_4_ (a) 0.03 g, (c) 0.05 g, and (e) 0.07 g, plots of ln(*C*_0_/*C*) *vs.* time (b) 0.03 g, (d) 0.05 g, and (f) 0.07 g under visible-light irradiation.

The photocatalytic activity of Rb_2_HgI_4_ nanostructures was studied by monitoring the degradation of anionic and cationic coloring agents as organic contaminants, such as acid black 1 (AB1), methyl orange (MO), methyl violet (MV), and rhodamine B (RhB) in an aqueous solution, under visible light (Osram light (150 W lamp produces 2600 lumens of light)) (Fig. 8). The spectral irradiance for the visible lamp was 400–780 nm according to the information provided by the manufacturer. Without Rb_2_HgI_4_ or light, almost no dyes were degraded after 90 min, revealing that the self-degradation part was insignificant. The degradation percentage (%*D*) was defined as follows:

2 %*D* = (*A*_0_ − *A*_t_)/*A*_0_ ×100

where *A*_t_ and *A*_0_ are the solution absorbance of the sample after and before degradation, respectively.^32^ The influence of various coloring agents and catalyst dosage was measured to obtain the optimum performance. Fig. 8a reveals the photocatalytic degradation of AB1, MO, MV, and RhB in the presence of 0.03 g Rb_2_HgI_4_. The Rb_2_HgI_4_ nanostructures can destroy these coloring agents by about 56.9%, 51.0%, 40.0%, and 36.1% after 90 min, respectively. The decolorization percentage of 0.05 g Rb_2_HgI_4_ are about 65.0%, 56.2%, 45.0%, and 41.1% for AB1, MO, MV, and RhB, respectively (Fig. 8c). Increasing the Rb_2_HgI_4_ dosage to 0.07 g enhanced the photocatalytic degradation to 72.1%, 63.1%, 55.1%, and 48.1%, respectively (Fig. 8e). Increasing the catalyst dosage is conducted in the space of the catalyst surface and enhances the dye adsorption on the catalyst surface.^33^ Besides, Rb_2_HgI_4_ nanostructures can destroy the anionic coloring agents better than the cationic ones. Hence, the highest performance was for AB1 (72.1%), and RhB (cationic dye) had the minimum yield. Table 3 shows the comparison of the photodegradation of different iodide compounds under visible and UV light. As demonstrated in this table, Rb_2_HgI_4_ can compete with other iodide compounds as a photocatalyst. We can propose Rb_2_HgI_4_ as a novel catalyst for the water purification process. Moreover, the possible reaction rate constants of the coloring agents were determined depending on the Langmuir–Hinshelwood mechanism.^28,34^

3 ln(*C*_0_/*C*) = *kt*

where *C*_0_ is the initial concentration of coloring agents; *C* is the concentration of coloring agents at *t* time; *k* is the pseudo-first order rate constant (min^−1^). The rate constant (*k*) was determined from the linear plot of ln(*C*_0_/*C*) *vs.* reaction time. As displayed in Fig. 8b, d, and f, better photocatalytic performance was achieved with a higher reaction rate constant.

**Table 3:** The photocatalytic activity of different iodide compounds

Sample	Highest decolorization (%)	Lowest decolorization (%)	Catalyst dosage (g)	Source of light	Ref.
Rb_2_HgI_4_ (sample 2)	72.1 (AB1)	48.1 (RhB)	0.07	Vis	This work
Tl_4_HgI_6_ nanostructures	76.9 (RhB)	48.9 (ThB^a^)	0.07	UV	38
Tl_4_Cdl_6_ nanostructures	85.7 (AB1)	49.1 (MB^bb^)	0.05	UV	29
CsPbl_3_ nanostructures	81.7 (MV)	33.0 (AB1)	0.07	Vis	33
Cu_2_CdI_4_/CuI nanocomposites	66.0 (MB)	29.1 (MO)	0.05	UV	41
Ag_2_CdI_4_ nanostructures	95.3 (RhB)	57.1 (AB1)	0.05	UV	42
TlCdI_3_ nanostructures	94.6 (MB)	27.0 (MO)	0.05	UV	43
Ag_2_ZnI_4_/AgI nanocomposites	89.3 (MO)	—	0.05	UV	44
Cu_2_ZnI_4_/ZnO nanocomposites	54.2 (MO)	—	0.05	UV	45

a Thymol blue.

b Methylene blue.

**Fig. 9:**
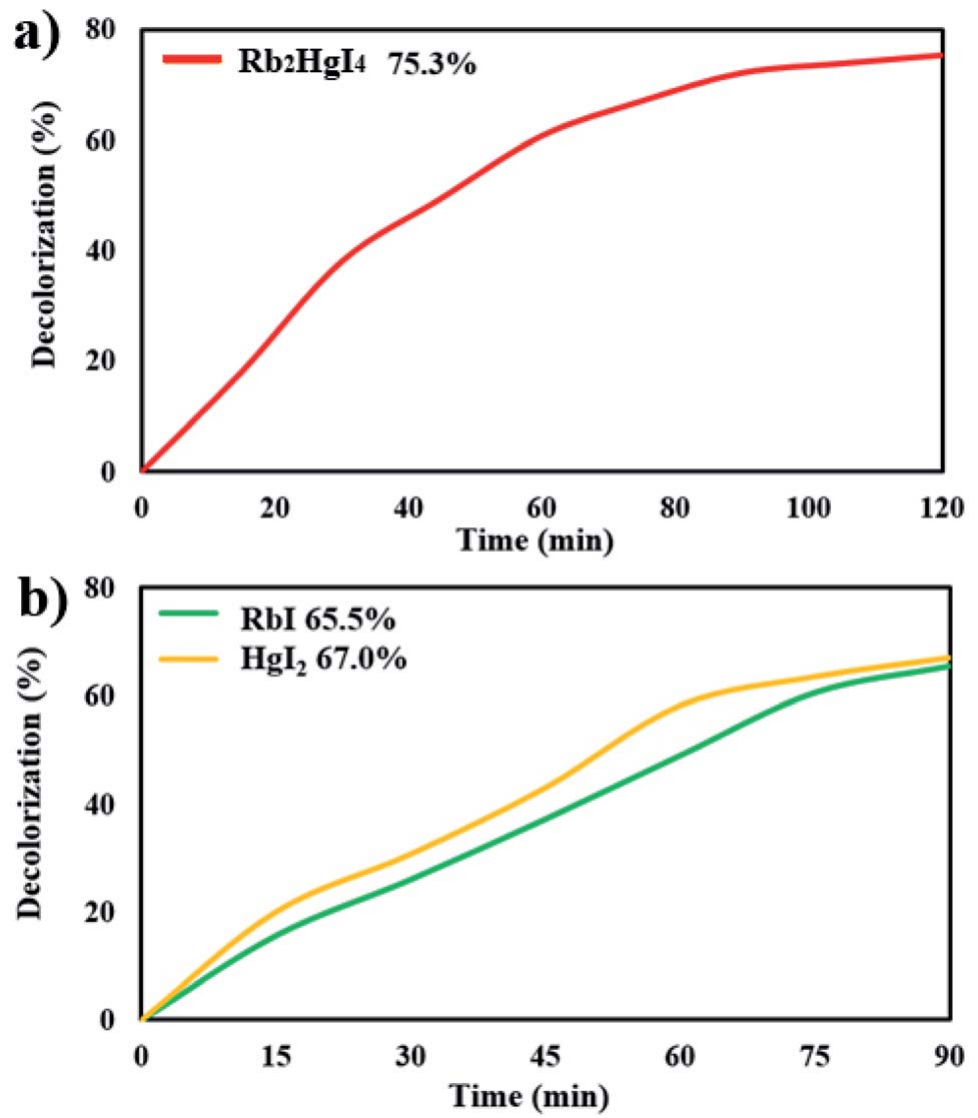
Photocatalytic degradation of (a) 0.07 g Rb_2_HgI_4_ after 120 min, (b) 0.07 g RbI and HgI_2_ after 90 min under visible light.

Fig. 9a shows the decolorization of AB1 under visible light after 120 min, and the percentage was obtained to be 75.3%. Increasing the time of the photocatalytic reaction improved the destruction by about 3%. The photocatalytic behavior of RbI and HgI_2_ was studied over AB1 under visible light, indicating 65.5% and 67.0% degradation, respectively (Fig. 9b). The result showed that the performance of Rb_2_HgI_4_ for the degradation of organic dye is higher than its precursors.

#### 3.2.1 Photocatalytic reaction mechanism

**Scheme 2:**
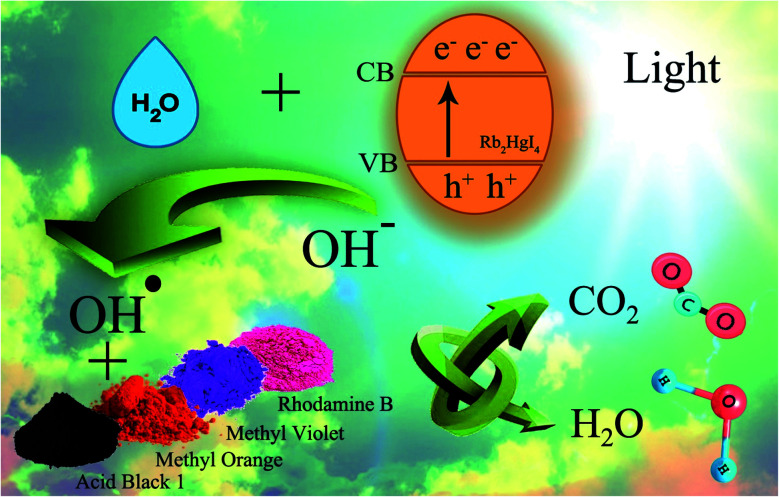
Schematic of the mechanism for the photocatalytic degradation of Rb_2_HgI_4_ nanostructures over different dyes.

Scheme 2 shows the schematic of the mechanism for the photocatalytic degradation of Rb_2_HgI_4_ nanostructures over different dyes. Multiple primary reactive species, including ^1^O_2_, H˙, HO˙, O_2_˙^−^, and h^+^ can be generated through the photocatalytic destruction procedures in semiconductors.^35^ The O_2_˙^−^ generation can restrict the photoinduced charge carrier recombination. HO˙ may be produced only in the e− → O_2_˙^−^ → H_2_O_2_ → OH˙ procedure. In addition, the hydroxyl radicals are produced through various reduction steps of superoxide anions in the process. As maintained in previous researches,^33^ the main reactive oxygen species (ROS) generated within the oxidation reactions and photocatalytic process are ^1^O_2_ and OH˙ radicals, respectively. Based on the mentioned concepts, we can propose that the generation possibility of O_2_˙^−^ should be much higher than the OH˙ production. Nonetheless, OH˙ is a strong, non-selective oxidizing agent that leads to the complete or incomplete mineralization of diverse organic mixtures. As mentioned in descriptions, the active species in the mechanism of organic dye degradation are O_2_˙^−^, ˙OH, and ^1^O_2_. There are literature reports indicating that the superoxide anion (O_2_˙^−^) plays the principal part, while the hydroxyl radical (˙OH) and singlet oxygen (^1^O_2_) play a minor part in the degradation of dyes.^36^ O_2_^−^˙ might be created *via* the reaction of O_2_ and/or ^1^O_2_ with e− species. The ^1^O_2_ might be produced *via* an h^+^ with O_2_^−^˙ species. The ˙OH might be generated with an h^+^ and H_2_O.^33^ Hence, O_2_^−^˙ could play the role of most basic active species in this study. We can perceive the photocatalytic mechanism for eliminating the organic pollutants as follows:^33,37,38^
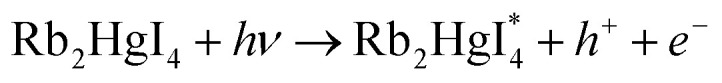
O_2_ + e^−^ → ˙O_2_^−^h^+^ + H_2_O → ˙OH + H^+^˙O_2_^−^+ e^−^+ H^+^ → HOO˙HOO˙ + H_2_O → ˙OH + H_2_O_2_2H^+^ + ˙O_2_^−^ → H_2_O_2_H_2_O_2_ + e^−^ → ˙OH + OH^−^OH^−^ + h^+^ → ˙OHH_2_O + h^+^ → ˙OH + H^+^˙O_2_^−^ or ˙OH + coloring agents → degradation sampleh^+^ + coloring agents → coloring agents^+^˙ → degradation sampleO_2_^−^˙ or ˙OH + coloring agents^+^˙ → degradation sample

## 4. Conclusions

Recently, photocatalysts based on semiconductors have gained significant attention as primary substances in the environmental remediation and water treatment. Nevertheless, most of them cannot show photocatalytic performance in the visible range because of their wide band gap. Furthermore, a high recombination rate is another crucial parameter that decreases the sunlight energy transformation performance. Here, we introduced a novel photocatalyst with a suitable band gap in the visible area. Rb_2_HgI_4_ nanostructures have been fabricated by the solid-state method at low temperatures (220 °C). The impact of various parameters, including surfactant types, time, and temperature of the reaction on the structure, purity, morphology, shape, and particle size was studied. The band gap of Rb_2_HgI_4_ was determined to be 2.6 eV, which makes this compound suitable for photocatalytic activity. The photocatalytic results revealed that Rb_2_HgI_4_ degraded acid black 1 by about 72.1%.

## Conflicts of interest

The authors declare that there are no conflicts of interest regarding the publication of this manuscript.

## Supplementary Material
